# Qualitative Traits and Antioxidant Properties of Blood Oranges Are Affected by the Genotype and the Climatic Conditions

**DOI:** 10.3390/foods13193137

**Published:** 2024-09-30

**Authors:** Giulia Modica, Pilar Legua, Stefano La Malfa, Alessandra Gentile, Alberto Continella

**Affiliations:** 1 Department of Agriculture, Food and Environment, University of Catania, 95123 Catania, Italy; giulia.modica@unict.it (G.M.); stefano.lamalfa@unict.it (S.L.M.); alessandra.gentile@unict.it (A.G.); 2 Plant Science and Microbiology Department, Miguel Hernández University, 03202 Alicante, Spain

**Keywords:** *Citrus sinensis*, nutraceutical compounds, anthocyanins, ascorbic acid, phenols

## Abstract

Blood oranges are increasingly cultivated worldwide as consumers become more aware of the health benefits of their nutraceutical properties and natural antioxidants, specifically polyphenols and anthocyanins. The amounts of these compounds in the fruit mostly depend on the cultivar, rootstock, maturity stage, and environmental conditions. This work focused on the study of the qualitative features of numerous blood orange cultivars grown in three different environments in Spain and Italy. The aim of the work was to investigate the accumulation of primary and secondary metabolites, including bioactive compounds, and to characterize fruit qualitative traits at the time of harvest. Simple sugars were identified and quantified by liquid chromatography and organic acids, polyphenols, and flavonoids by spectrophotometric analysis. The antioxidant potential of the juice was assessed by ABTS, DPPH, and FRAP assays. Cultivation area affected juice color, with Moro and T. Ippolito being the varieties with the highest pigmentation. The cultivation area also determined the pattern of primary and secondary metabolite accumulation in the Tarocco lines. Furthermore, the antioxidant potential was influenced by the diverse environments. Principal Component Analysis highlighted three clusters, two overlapping clusters for the varieties grown in the two Spanish plots and a third clearly separated cluster for the genotypes grown in Italy. This study provides novel knowledge on primary and secondary metabolite accumulation in blood oranges, elucidating the role of genotype and environmental conditions on fruit quality.

## 1. Introduction

Blood sweet oranges [*Citrus sinensis* (L.) Osbeck] differs from blond oranges mainly by the presence of red color in the peel and flesh of the fruit. The pigmentation of blood oranges is associated with the presence of anthocyanins, water-soluble pigments that belong to the larger family of phenols. The amount of these pigments is remarkably influenced by several factors, including cultivar, rootstocks, maturity stage, site of cultivation, and climatic conditions [1,2,3,4].

The consumption of blood oranges has increased in recent years, especially in Europe [5], but they are cultivated only in a few citrus-producing countries, especially in the Mediterranean area, i.e., Italy and Spain, where climatic conditions favor the biosynthesis of the red pigment [5,6,7,8,9]. In this context, in Spain, the main blond-orange-producing country in Europe, the cultivation of blood oranges has also recently increased [7], although historically blood oranges were grown mainly in Italy [10].

The most cultivated variety in Italy is Tarocco, characterized by a number of lines with different qualitative characteristics and maturation periods, followed by the Moro and Sanguinello varieties [11,12]. Tarocco oranges are appreciated for their low sugar/acid ratios and their peelability; Moro cv. oranges show the reddest color of the peel and their juice is rich in biochemical compounds, especially hydroxycinnamic acids and anthocyanins [10,13,14]. In Spain, the most widespread blood orange cultivar is Sanguinelli, a spontaneous mutation of the Doble Fina variety [9]. Colorimetric analysis of citrus juice, and especially, the citrus color index, is a useful tool for monitoring fruit maturity in the citrus industry, and especially, pigmentation levels in blood oranges [9,15,16].

Among primary metabolites, sugars and organic acids, predominantly citric acid, are distinguishable in citrus fruits with their characteristic sour taste. The sweetness and distinctive palatability of citrus fruit depend on the content of carbohydrates, the main soluble components in the pulp of citrus fruit (75–80%), and the ratio between sugar and organic acids in the juice [16]. As is well known, citric acid is the major organic acid in oranges, as previously reported both in blond and pigmented oranges [9,17,18]. Blood oranges exhibit generally more organic acids than blond oranges at harvest [19]. Among bioactive compounds, vitamin C is an important nutraceutical compound in blood oranges because of its antioxidant activity [7]. Secondary metabolites, including flavonoids, phenolic acids, and terpenes, are beneficial to human health, regulating physiological activities and having antibacterial and anticancer properties [20,21]. The antioxidant potential of the phytochemical compounds has been mostly studied due to their ability to respond to oxidation processes preventing diseases related to oxidative damage in the human body [22,23]. Different categories of antioxidant compounds are considered natural sources, and for this reason, the measurement of antioxidant capacity is very complex because no single method is able to fully represent the natural reactions that occur in vivo [24]. Previous studies reported that the ABTS test was mostly used to evaluate the antioxidant potential of flavonoids (flavones, flavanones, and flavonols) and hydroxycinnamic acids (including ferulic acid and p-coumaric acid). However, other methodologies, i.e., DPPH and FRAP assay, are also necessary to better understand the influence of several nutraceutical compounds [25,26]. Previous studies highlighted that a decisive role in the antioxidant potential is played by the polyphenols contained in blood orange juice, particularly the anthocyanins, and it was also noted that the variability in the composition of the different phenols is genotype dependent [4,21,27].

However, there is a lack of research on the comprehensive investigation of how the synthesis of the abovementioned primary and secondary metabolites is affected by genotype and environmental conditions. In such a context, in this study, the synthesis of primary and secondary metabolites in orange juice was investigated at three different sites in order to evaluate the roles of environmental and genotype in blood orange fruit qualitative traits, the accumulation of sugars, organic acids, phenols and anthocyanins, and antioxidant activity.

## 2. Materials and Methods

### 2.1. Plots of Cultivation and Plant Material

The experiment on the evolution of the maturation of blood oranges was carried out in three different sites situated in Spain and Italy. Plot 1 was located in Lentini, Italy (37°17′04″ N; 14°53′16″ E), Plot 2 experimental field was in Orihuela, Spain (38°03′57″ N; 0°58′58″ W), and Plot 3 in Huelva, Spain (37°25′05″ N; 7°02′54″ W). The plants spaced 5 m × 3 m apart, were cultivated with the same agronomical practices.

**Figure 1:**
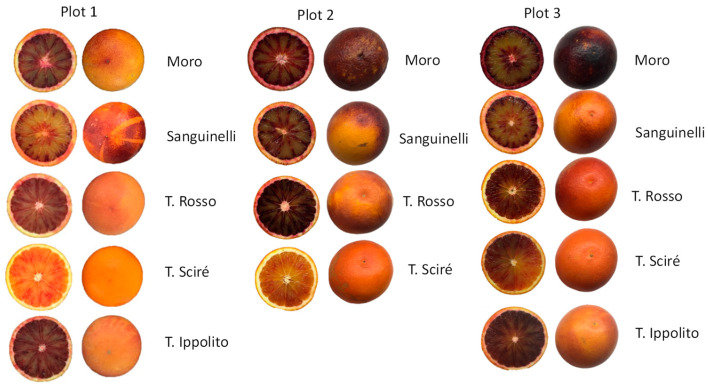
Flesh and peel of blood orange fruits cultivated in plots 1, 2, and 3.

The cultivars evaluated are reported in Figure 1. Four varieties were present in all three plots (Tarocco Rosso, Tarocco Scirè, Moro, and Sanguinelli), while a fifth cultivar was sampled only in plots 1 and 3 (Tarocco Ippolito). All of the cultivars are classified as mid-season maturing oranges. The fruits in each plot were harvested in mid-February, 275 days after full bloom (DAFB). For each cultivar, four replicated 5-to-7-year-old trees grafted on Carrizo citrange rootstock and in good phytosanitary status were selected for sampling. In order to characterize the environment of the three plots, data of the maximum, mean, and minimum temperature and of the monthly rainfall of each plot (Figure S1) were recorded from 2018 to 2023. Data were provided by weather stations located near the plots managed by Servizio Informativo Agrometeorologico Siciliano, SIAS (http://www.sias.regione.sicilia.it/ accessed on 15 July 2024) and by Sistema de Informacion Agoclimatica para el Regadio, SIAR (https://servicio.mapa.gob.es/websiar/ accessed on 15 July 2024).

### 2.2. Morphological and Physico-Chemical Parameter Determination

Thirty representative pooled fruits from each variety for each plot were sampled, collected, individually weighed, measured, and then used for juice extraction. Juice color was recorded in each fruit using a Minolta CR-400 chroma-meter (Minolta Corp., Osaka, Japan) and the results were expressed as a citrus color index (CCI = a*1000/L*b), calculated as reported by Caruso et al. [28]. Fruit juice was extracted with a commercial juice extractor (Kenwood Citrus Juicer JE290, Havant, Hampshire, UK). Three juice samples, from the pooled juice of 5 fruits from 3 replicates, were used for chemical analyses. Juice was weighed and expressed as a percentage of the total fruit weight. The Total Soluble Solids (TSS) content was determined using a digital refractometer (Atago Co., Ltd., model PR-32 α, Tokyo, Japan), with the results expressed as °Brix. Titratable acidity (TA) was determined by potentiometric titration (Hach, TitraLab AT1000 Company, Loveland, CO, USA) of the juice with 0.1 N NaOH above pH 8.1 according to the AOAC method (AOAC, Washington, DC, USA, 1995), with the results expressed as g L^−1^ of citric acid equivalent. Ripening index (RI) was calculated as the ratio between TSS and TA.

### 2.3. Determination of Sugars and Organic Acids

Individual organic acids and sugars were quantified using three juice samples for each variety as described by a previous study [29]. Briefly, one milliliter of the centrifuged juice was passed through a 0.45 μm Millipore filter and then injected into a Hewlett-Packard series 1100 (Hewlett-Packard, Wilmington, DE, USA) high-performance liquid chromatography (HPLC) system. The elution system consisted of 0.1% phosphoric acid with a flow rate of 0.5 mL min^−1^. Organic acids were separated on a Supelcogel TM C-610H column (30 cm × 7.8 mm i.d., Supelco, Bellefonte, PA, USA) and Supelguard column (5 cm × 4.6 mm, Supelco, Inc., Bellefonte, PA, USA), and detected using a diode-array detector set up at 210 nm. For sugar analyses, the same HPLC equipment, elution system, flow rate, and columns were used. The detection of sugars was performed using a refractive index detector (HP 1100 series, G1362A Agilent, Santa Clara, CA, USA). Standard curves for pure standards of organic acids (citric and ascorbic acids) and sugars (glucose, fructose, and sucrose) (Sigma, Poole, Dorset, UK) were used for identification and quantification. Results for both organic acids and sugars were expressed as concentrations g 100 mL^−1^ and the samples were determined in triplicate.

### 2.4. Polyphenols, Total Anthocyanins Content and Antioxidant Activity

Total polyphenol content (mg L^−1^) was quantified using Folin–Ciocalteu reagent [30]. Briefly, for each sample, 2 g of flesh tissue was homogenized in 5 mL of MeOH/water (80:20 *v*/*v*) + 2 mM NaF and then centrifuged at 15,000 rpm for 20 min. Absorption was measured at 760 nm using a spectrophotometer (NanoDrop 2000, Thermo Scientific, Waltham, MA, USA).

Total anthocyanin content was determined spectrophotometrically by the pH differential method previously described [31]. The absorbance was measured using a spectrophotometer (NanoDrop 2000, Thermo Scientific) at 510 and 700 nm in buffers at pH 1.0 and 4.5, respectively. The results were expressed as mg of cyanidin-3-glucoside equivalents per mg L^−1^.

The antioxidant activity (mM Trolox) was determined by different methods: the ABTS^+^ [2,2-azinobis-(3-ethylbenzothiazoline-6-sulfonic acid)] method and the DPPH radical method (2,2-diphenyl-1-picrylhydrazyl) according to Modica et al. [4], and the FRAP assays (ferric reducing antioxidant power), conducted as previously described [32].

### 2.5. Statistical Analyses

Statistical analyses were performed using STATISTICA 6.0 (Statsoft Inc., Tulsa, OK, USA). The method used to discriminate among the means (multiple range test) was Fisher’s least significant difference (LSD) procedure at a 95.0% confidence level. Principal component analysis (PCA) was performed using R software (v. 4.3.1) computing the “prcomp” function of the package ‘tidyverse’ [33]. The results were represented using the package ‘ggplot2’ [34].

## 3. Results and Discussion

### 3.1. Climatic Site Characterization

The site of cultivation and, specifically, the temperatures registered in the field assume a pivotal role in the ripening process of citrus fruit. Blood oranges are characterized by a peculiarity in the ripening process in that the pigmentation of both the peel and the pulp is closely connected to the environmental conditions that occur during the ripening period, which takes place in the autumn–winter. It is reported that in order to obtain oranges with high pigmentation, it is necessary to have temperature excursions wide enough to stimulate the synthesis of anthocyanins in this period and, at the same time, temperatures high enough to allow the accumulation of sugars [2,4,35]. Other authors hypothesize that, given the same cultivar and rootstock, there are certain temperatures recorded in the field, specifically in the range between 5 and 10 °C, that particularly stimulate anthocyanin biosynthesis [4], while high temperatures play a direct effect on internal ripening of citrus fruit [36].

From the climatic data of the temperatures recorded in the last 5 years in the three plots under study, a similar high thermal excursion was observed, with values of over 20 °C between the maxima and minima registered from November to February, the months in which anthocyanins accumulate in blood oranges. In detail, in all plots, the highest maximum temperatures recorded were in the range 20–25 °C and the lowest minimum temperatures were between 0 and 5 °C (Figure S1). The climatic conditions were, therefore, suitable for the cultivation of blood oranges. Specifically, the cold temperatures necessary for anthocyanin biosynthesis occurred in the three plots, and, at the same time, the temperature variations between the maximum and minimum were wide.

Previous researchers [37] discovered in blood oranges high levels of expression of the Ruby gene, which is involved in the biosynthesis of anthocyanins. However, the expression of genes involved in anthocyanin synthesis is influenced by field climatic conditions and also by agronomic techniques, including the choice of the rootstock [2,3,4,6]. Several studies have focused on the role of temperature in the activation of anthocyanin biosynthesis pre- and post-harvest [4,38,39,40]. A multiomics approach is necessary for further research to better understand the biosynthesis of individual metabolites involved in blood orange pigmentation under specific environmental conditions.

### 3.2. Morphological and Biochemical Analyses

Previous research noticed that blond and blood orange cultivars exhibited TSS values ranging between 11 and 15 °Brix, while the characteristic taste of the citrus juice is due to the organic acids, with TAs ranging between 11 and 14 g L^−1^ of citric acid, depending on maturity, environmental conditions, and agronomical practices [9,35]. In accordance with this, in plot 1, the highest TSS content was observed in Tarocco Sciré (13.6 °Brix), while the lowest value was recorded in Sanguinelli (11.0 °Brix). Indeed, the content of TA ranged from 7.8 g L^−1^ in Tarocco Ippolito to 14.0 g L^−1^ in Sanguinelli, the latter reaching the lowest RI value among all cultivars (Table 1). In plot 2, T. Rosso reached the highest value of TSS (15.4 °Brix), while Sanguinelli displayed the lowest TSS (12.4 °Brix) and highest TA (Table 1), reaching again the lowest RI value. In plot 3, T. Rosso was the cultivar with the highest TSS, while Moro showed the lowest value (11.6 °Brix) in accordance with the results recorded in a previous work [41].

Regarding the TSS/TA ratio, the European Commission determined that the minimum sugar/acid ratio for the commercialization of blood or navel oranges is 6.5 [42]. Generally, the ripening indices (RIs) of blood oranges usually do not reach the higher values recorded in blond oranges because, at the ripening stage, acids decrease more slowly in blood oranges than in blond oranges [9,19]. Overall, T. Ippolito showed the highest RI in the two sampled plots, even if the TSS content was not the highest, Conversely, the fruit of cv. Sanguinelli exhibited the highest TA, confirming the findings of the aforementioned study [41], and this cultivar was also determined to have the lowest RI in all environmental conditions.

Juice percentage values were in line with the standard values reported for the studied cultivar, as also found by other authors [43,44].

Color determination is a non-invasive method for the determination of the maturation stage and the qualitative features of citrus fruit and it is generally expressed by an index called the citrus color index (CCI) [6]. An interesting behavior was observed regarding juice color, as Moro showed the reddest color of all the juices in plots 1 and 2, while Sanguinelli exhibited the greatest pigmentation in plot 3 (Table 1). This confirms that even if the biosynthesis and accumulation of anthocyanins are related to the genotype, the interaction with the environmental conditions may play a prevalent role [4,6].

**Figure 2:**
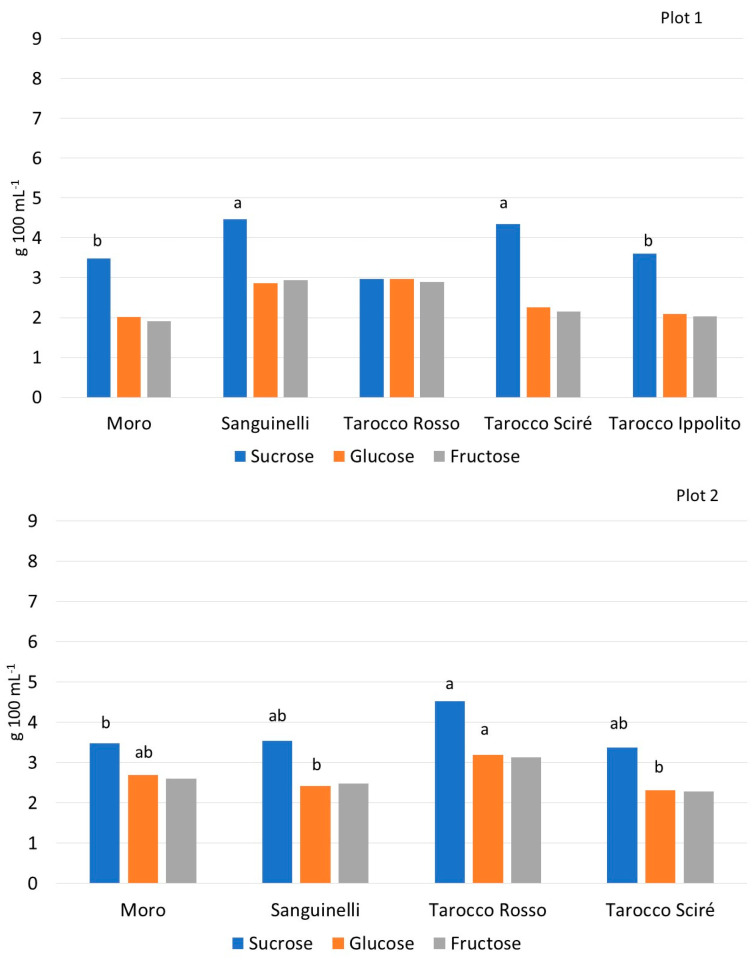

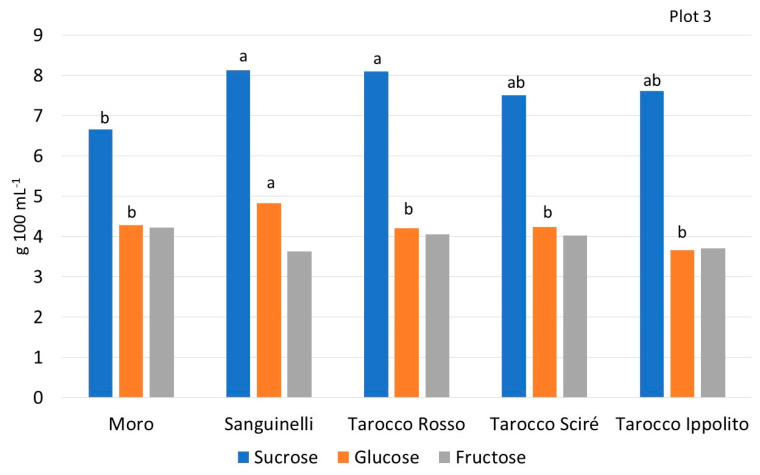
Glucose, fructose, and sucrose (mg 100 L^−1^) content of the fruits of different blood orange cultivars cultivated in three different plots. Values without letters have no significant differences according to Fisher’s LSD procedure at a 95 % confidence level.

Analyzing the accumulation of individual sugars in the three plots, a different pattern was recorded in each environment (Figure 2). Generally, orange juice sucrose, glucose, and fructose account for about 80% of the total soluble solids, and the sweetness of the juice depends on their total amount. The sucrose:glucose:fructose ratio is usually approximately 2:1:1 [16,18,45], and this proportion was confirmed by the HPLC analyses carried out on the fruits of all cultivars sampled in the three plots (Figure 2).

In plot 1, the highest content of glucose was recorded in T. Rosso (3.0 g 100 mL^−1^), while the lowest was observed in Moro (2.0 g 100 mL^−1^), although no statistical difference between the genotypes was observed; the quantities of fructose ranged from 1.9 g 100 mL^−1^ (Moro) to 2.9 g 100 mL^−1^ (Sanguinelli). Meanwhile, the sucrose content was significantly the highest in Sanguinelli (4.5 g 100 mL^−1^), while the smallest quantity was found in T. Rosso (3.0 g 100 mL^−1^). In plot 2, T. Rosso displayed the highest glucose and sucrose values among all cultivars, while the amount of fructose was similar in all genotypes. In plot 3, Moro showed the lowest quantity of sucrose, while Sanguinelli exhibited the highest amount of both glucose and sucrose. No statistical differences were recorded for the quantities of fructose between the genotypes. Overall, these results were in accordance with previous studies on blood orange cultivars carried out by Habibi et al. [46] and Morales et al. [47]. In plot 3, the genotypes accumulated a higher amount of the single sugars analyzed than the quantities synthesized in the other environmental conditions.

Blood oranges generally exhibit a higher amount of organic acids than blond oranges and citric acid is the prevalent compound [19].

**Figure 3:**
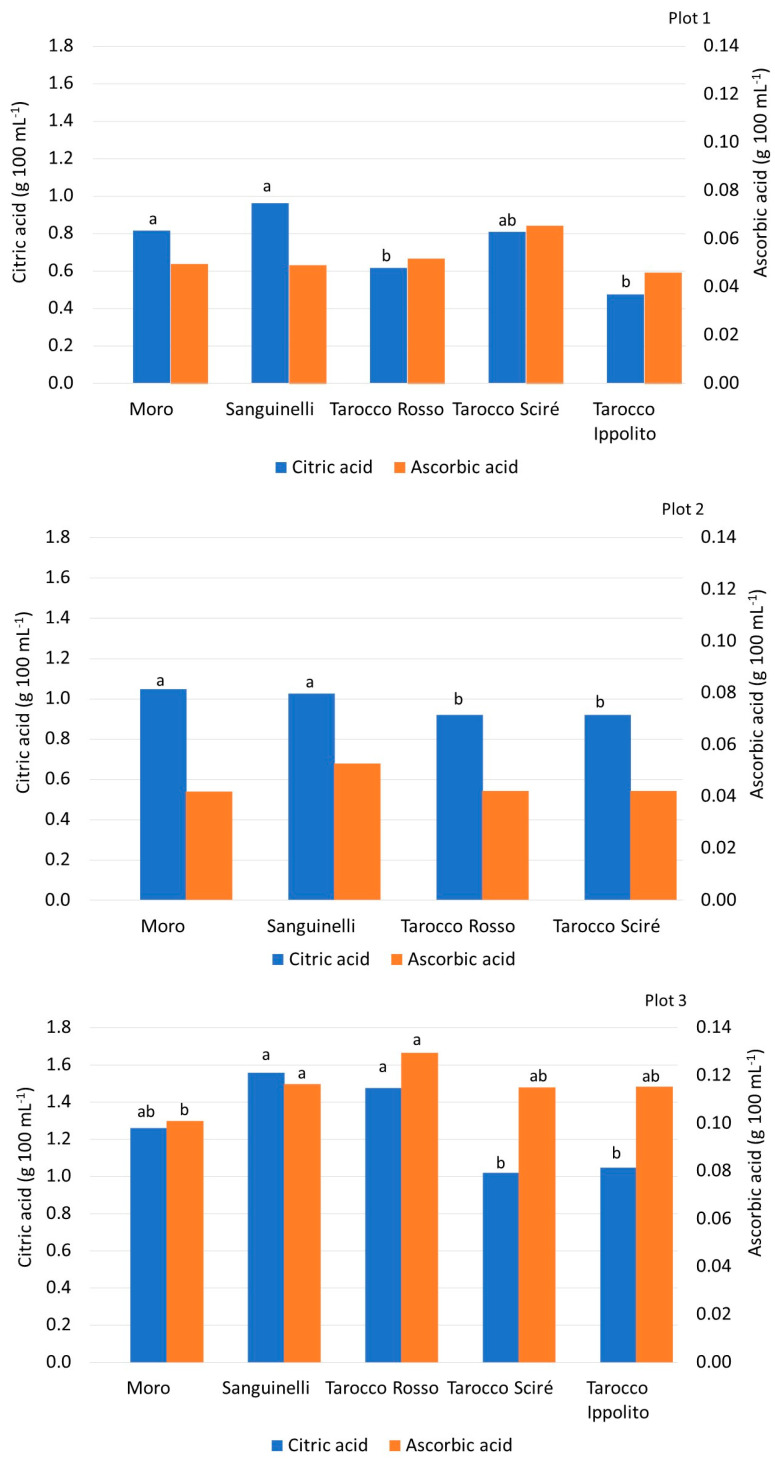
Content of citric and ascorbic acids (g 100 mL^−1^) of the fruits of different blood orange cultivars cultivated in three different plots. Values without letters have no significant differences according to Fisher’s LSD procedure at a 95% confidence level.

In plot 1, Sanguinelli and Moro presented the highest levels of citric acid, while T. Ippolito and T. Rosso had the lowest amount (Figure 3). In plot 2, citric acid levels ranged from 0.91 (T. Scirè and T. Rosso) to 1.0 g 100 mL^−1^ (Moro). In plot 3, T. Sciré had the lowest amount of citric acid, while Sanguinelli showed the highest. The range of citric acid quantified in all genotypes confirmed the results reported by previous researchers for pigmented oranges [9,18]. Ascorbic acid plays an important role in human health due to its antioxidant activity and it is a nutraceutical compound with higher amounts reported in blood oranges than in blond oranges [7,48]. No statistical difference was observed between the studied genotypes in plots 1 and 2. In plot 3, an overall higher amount of ascorbic acid than in the other plots was detected and T. Rosso (0.13 g 100 mL^−1^) exhibited the highest content (Figure 3), while Moro showed the lowest. This is in contrast to previous studies, which reported that Moro showed higher amounts of ascorbic acid than that measured in Tarocco cultivars [9,49].

### 3.3. Polyphenols and Anthocyanins Content and Antioxidant Activity

**Figure 4:**
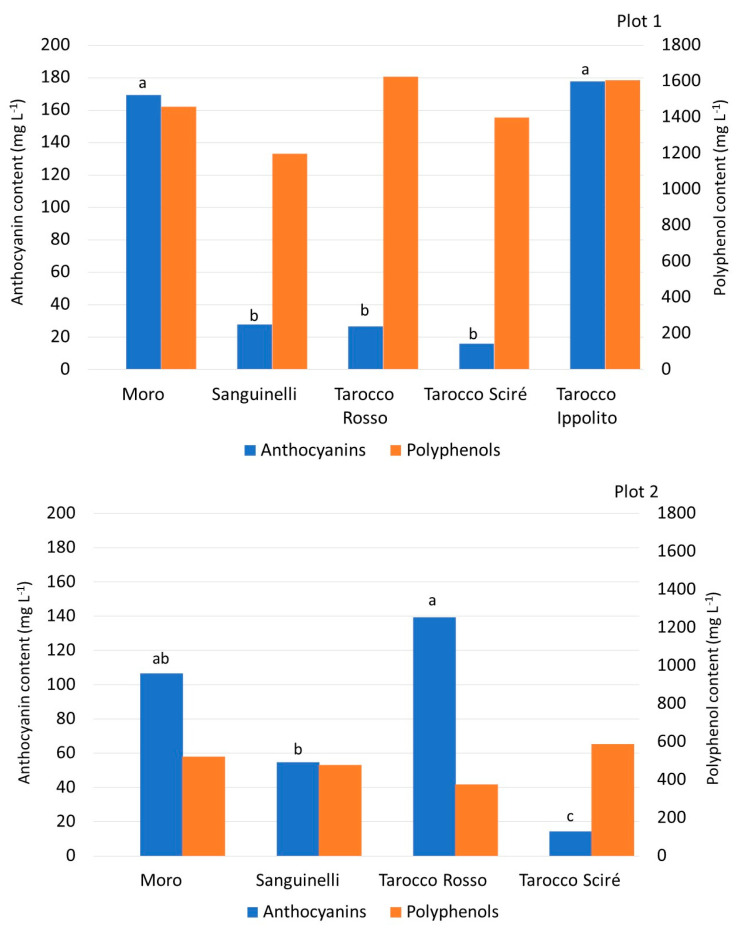

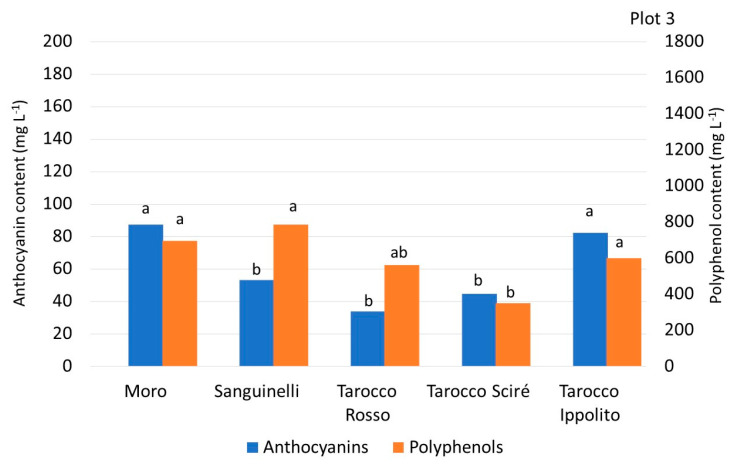
Total anthocyanin and polyphenols content (mg L^−1^) of the fruit sampled at harvest time from different blood orange cultivars cultivated in three different plots. Values without letters have no significant differences according to Fisher’s LSD procedure at a 95% confidence level.

Fruit qualitative traits, including pigmentation, may depend on the environment; actually, climatic conditions affect mostly anthocyanin biosynthesis during fruit ripening, and low night temperatures are essential for intense color formation [50]. Blood orange fruits synthesize several biochemical compounds, including phenol and anthocyanin components in the pulp and peel. Indeed, the bioactive compounds in the juice, i.e., ascorbic acid, hydroxycinnamic acids, and flavonoids, have been considered to play an important qualitative and healthy role, and have received increasing consumer awareness for their antioxidant activity [4,7,41,48]. Moro and T. Ippolito reached the highest anthocyanin content in plots 1 and 3, while Moro and T. Rosso had the highest amount in plot 2 (Figure 4).

Previous researchers reported that the total anthocyanin content in Moro juice was greater than the amount detected in T. Rosso and in the Italian variety Sanguinello [44,47]. In all three studied plots, Sanguinelli did not show high amounts of anthocyanins. This is in contrast to other studies, which reported the highest pigmentation levels in Sanguinelli, followed by Moro [9,41]. In addition, among Tarocco lines, only in plot 2, was a statistical difference between T. Rosso and T. Scirè observed, while the amounts of anthocyanins were similar in plots 1 and 3, in accordance with the results of Forner-Giner et al. [41].

Regarding the polyphenol content, on the whole, plot 1 exhibited the highest content of polyphenols with values of about twice those detected in the other two plots (Figure 4). No statistical difference was noted between the studied cultivars in plots 1 and 2, while, in plot 3, T. Scirè showed the lowest amount of phenols and Sanguinelli and Moro the highest amounts (784.1 and 694.3 mg L^−1^, respectively). In accordance with a previous study [48], the present investigation found that the quantity of polyphenols measured in Moro depended on the anthocyanin content.

**Figure 5:**
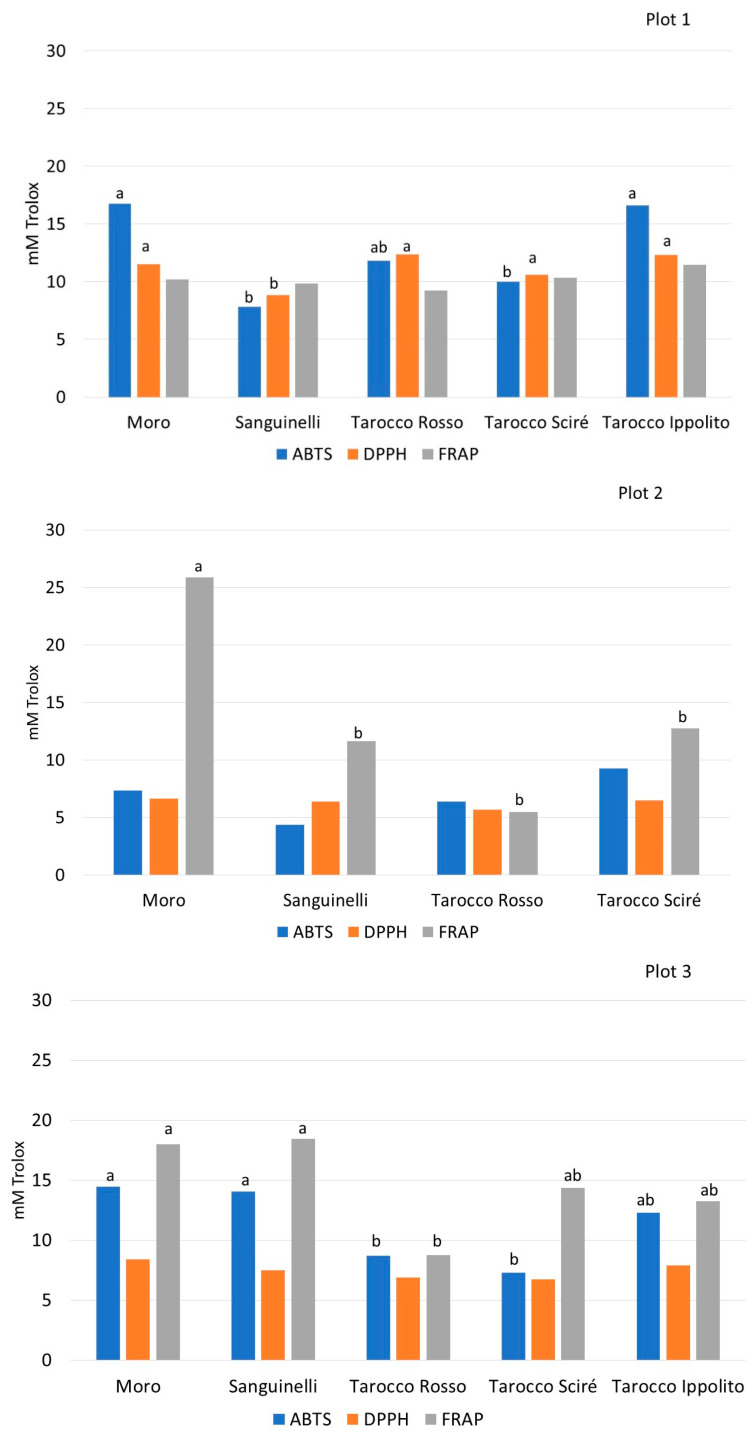
Antioxidant potential (mM Trolox) of the fruit sampled at harvest time from different blood orange cultivars cultivated in three different plots. Values without letters have no significant differences according to Fisher’s LSD procedure at a 95% confidence level.

Fruit tree crops biosynthesize primary and secondary metabolites to regulate different physiological mechanisms involving the activation of antioxidant enzymatic activities during plant growth and under biotic and abiotic stresses [51]. Especially in citrus, the quantity of phenols in the flesh plays an important role in the antioxidant activity of the juice [4,27,52]. In plot 1, Moro, T. Ippolito, and T. Rosso showed the highest ABTS and DPPH values, while Sanguinelli had the lowest antioxidant activity; the FRAP test did not disclose any significant difference between the sampled varieties (Figure 5). In plot 2, Moro exhibited the highest FRAP values, and no statistical difference was detected using the ABTS and DPPH methodologies. In plot 3, Sanguinelli, Moro, and T. Ippolito had the highest antioxidant potential measured using ABTS and FRAP tests. T. Sciré exhibited similar FRAP values (Figure 5).

The measurement of the antioxidant capacity is very complex because no single method is able to fully represent the natural reactions that occur in vivo [24], however, the methodologies employed are realistic and each test is able to measure the antioxidant activity in order to better understand the influence of bioactive compounds [25,26]. Under the conditions studied, the effects of both genotype and environment on the accumulation of the different components and their antioxidant activity were discernible.

**Figure 6:**
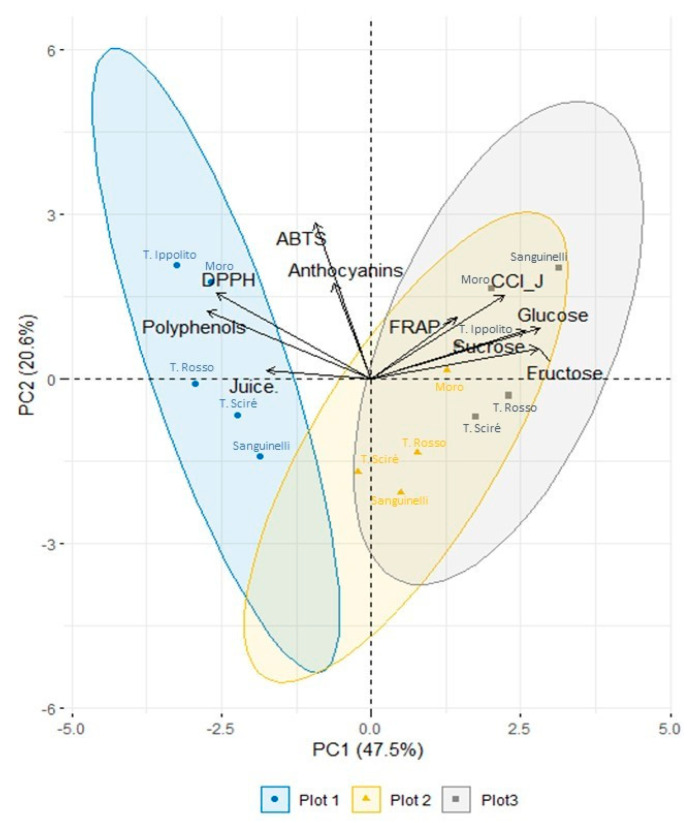
Principal component analysis (PCA) showing the distribution of the several genotypes cultivated in the plots. The parameters measured were Juice percentage (Juice), juice Citrus Colour Index (CCI_J), Sucrose, Fructose, Glucose, total anthocyanins (Anthocyanins), Total polyphenols (Polyphenols), ABTS, DPPH, and FRAP.

The results of the Principal Component Analysis (PCA) showed that the first two main components explained 68.15% of the total variation taking into account the qualitative and nutraceutical features of the fruits (Figure 6). PC1 (47.55% of variance explained) was positively correlated mainly with glucose (0.408), fructose (0.373), and sucrose (0.404). However, it was negatively correlated mainly with polyphenols and DPPH analyses. PC2 (20.60% of variance explained) was positively correlated mainly with the variables ABTS (0.627), DPPH (0.345), anthocyanins, and juice CCI (0.382 and 0.337, respectively). The results showed that Sanguinelli cultivated in plot 3 had the highest correlation with PC1 (0.384), followed by T. Rosso (0.281) due to the accumulation of sugars in the juice (Figure 3). It was noticed that T. Ippolito and T. Rosso cultivated in plot 1 were negatively related to PC1 (−0.400 and −0.361, respectively). Fruits of T. Ippolito (0.389) and Moro (0.328) cultivated in plot 1, and Sanguinelli (0.378) in plot 3, had the highest correlation with PC2 due to the highest juice pigmentation (Figure 1) and the antioxidant potential expressed by ABTS assay. Sanguinelli cultivated in plot 2 was negatively correlated with PC2 (−0.388), indicating the effective impact of the environment.

Overall, three clusters emerged, each one including the varieties of each plot. The two clusters of plots 2 and 3, representing the two Spanish sites, overlapped, while a clear separation was observed for the genotypes cultivated in the Italian site (plot 1). All qualitative and nutraceutical traits of the fruit, including primary and secondary metabolites in the juice, were shown to be involved in the PCA. It is important to consider that specific environmental conditions, especially temperatures, are crucial for the biosynthesis of anthocyanins [53,54,55]. In this context, the production of blood oranges is typical of Eastern Sicily and, in particular close to Mount Etna, where the minimum temperature is reported to affect pigment biosynthesis [4,6].

## 4. Conclusions

The recent growing interest in blood oranges is due to their natural antioxidants and bioactive compounds, such as polyphenols, including anthocyanins, and higher amounts of ascorbic acid. In this work, the effect of environmental conditions on the primary and secondary metabolites of different blood orange varieties was demonstrated. Different accumulations of sugars and organic acids were observed in the three plots studied, with the genotypes in plot 3 accumulating the highest content of single sugars and ascorbic acid. Regarding pigmentation, Moro and T. Ippolito reached the highest anthocyanin content in plots 1 and 3, while Moro and T. Rosso had the highest amounts in plot 2, supporting the prevalent role of the genotype in the pigmentation of blood oranges. Furthermore, the effects of the environment on juice biochemical compounds and antioxidant activity were confirmed. All qualitative traits and nutraceutical compounds analyzed by PCA showed a separation of plot 1 (varieties cultivated in Italy) from plots 2 and 3 (varieties cultivated in Spain), with an overlapping of the latter two plots. Overall, Moro and Sanguinelli confirm their suitability for juice extraction due to the high pigmentation, with Tarocco lines being more adaptable for fresh consumption.

## Figures and Tables

**Table 1:** Quantitative and qualitative characteristics of the fruits of different blood orange cultivars cultivated in three different plots. TSS: total soluble solids (°Brix); TA: titratable acidity (g L^−1^); RI: TSS/TA; Juice: percentage of juice (%); CI: juice color index (CI).

Plot 1	TSS (°Brix)	TA (g L^−1^)	RI (TSS/TA)	Juice (%)	CI
Moro	13.4 ± 0.46 a	13.4 ± 0.28 ab	10.0 ± 0.02 b	51.1 ± 2.92 c	71.2 ± 1.75 a
Sanguinelli	11.0 ± 0.56 b	14.0 ± 0.85 a	7.8 ± 0.03 c	53.4 ± 1.17 b	16.7 ± 7.72 b
Tarocco Rosso	12.9 ± 0.21 b	12.5 ± 0.54 b	10.3 ± 0.02 b	52.1 ± 1.98 c	15.0 ± 2.68 b
Tarocco Sciré	13.6 ± 0.49 a	12.4 ± 1.46 b	11.0 ± 0.12 b	54.7 ± 054 ab	9.7 ± 1.22 c
Tarocco Ippolito	12.5 ± 0.25 ab	7.8 ± 0.20 c	16.0 ± 0.02 a	57.3 ± 3.98 a	63.8 ± 20.49 a
**Plot 2**					
Moro	14.4 ± 0.12 b	13.8 ± 0.66 a	10.4 ± 0.03 b	48.6 ± 1.00 a	250.5 ± 14.62 a
Sanguinelli	12.5 ± 0.38 c	14.1 ± 0.28 a	8.9 ± 0.03 c	44.6 ± 3.20 b	92.7 ± 15.13 b
Tarocco Rosso	15.3 ± 0.17 a	12.3 ± 0.83 b	12.4 ± 0.06 a	47.6 ± 3.79 ab	72.2 ± 5.57 b
Tarocco Sciré	13.5 ± 0.13 c	12.1 ± 0.39 b	12.5 ± 0.05 a	47.6 ± 5.23 ab	27.3 ± 1.41 c
**Plot 3**					
Moro	11.6 ± 0.17 c	13.2 ± 0.92 b	8.8 ± 0.11 c	47.0 ± 2.54 b	162 ± 8.77 ab
Sanguinelli	12.8 ± 0.16 b	16.3 ± 0.75 a	7.8 ± 0.10 c	44.0 ± 6.62 c	333.5 ± 11.12 a
Tarocco Rosso	15.9 ± 0.65 a	15.9 ± 0.64 a	10.0 ± 0.10 b	48.9 ± 23.68 ab	153.7 ± 4.75 ab
Tarocco Sciré	12.1 ± 0.72 b	11.8 ± 0.78 c	10.3 ± 0.15 b	58.7 ± 4.18 a	48.4 ± 1.43 c
Tarocco Ippolito	12.0 ± 0.76 b	10.9 ± 0.92 c	11.0 ± 0.17 a	49.2 ± 2.61 ab	103.5 ± 4.21 b

Values with letters within the same column are not significantly different according to Fisher’s LSD procedure at a 95% confidence level (*n* = 30).

## Data Availability

The original contributions presented in the study are included in the article/Supplementary Materials, further inquiries can be directed to the corresponding authors.

## Supplementary Materials

The following supporting information can be downloaded at: https://www.mdpi.com/article/10.3390/foods13193137/s1, Figure S1: Monthly minimum (blue, °C), mean (grey, °C), and maximum (orange, °C) air temperature and rainfall (yellow, mm) registered in the three different plots from 2018 to 2023.
