# Supplementary material for: Qualitative Traits and Antioxidant Properties of Blood Oranges Are Affected by the Genotype and the Climatic Conditions
Source: Foods. 2024 Sep 30;13(19):3137. doi: 10.3390/foods13193137 (PMC11482589; doi:10.3390/foods13193137)
Supplement: Supplementary file 1 [file foods-13-03137-s001.zip › foods-3233391-supplementary.pdf]

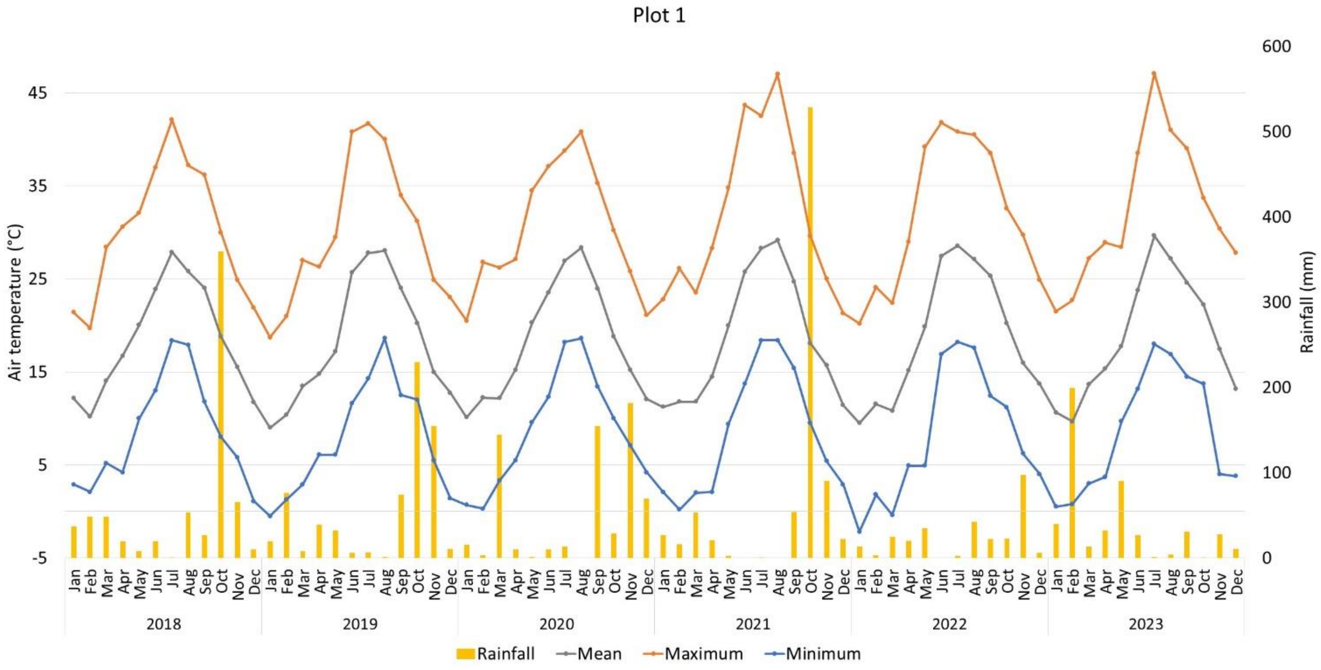
Plot 1
Air temperature (°C)
Rainfall (mm)
45
35
25
15
5
-5
600
500
400
300
200
100
0
Jan Feb Mar Apr May Jun Jul Aug Sep Oct Nov Dec
2018
2019
2020
2021
2022
2023
Rainfall
Mean
Maximum
Minimum

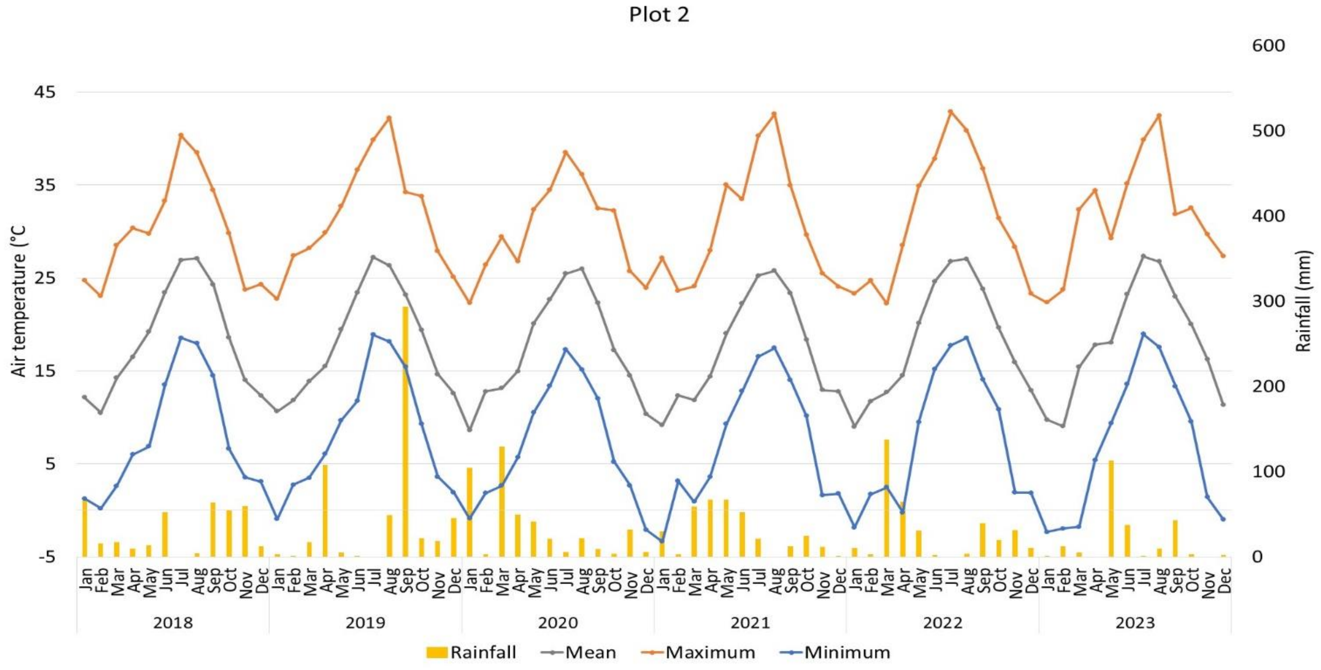
Plot 2
Air temperature (°C
Rainfall (mm)
45
35
25
15
5
-5
600
500
400
300
200
100
0
Jan Feb Mar Apr May Jun Jul Aug Sep Oct Nov Dec
2018
2019
2020
2021
2022
2023
Rainfall
Mean
Maximum
Minimum

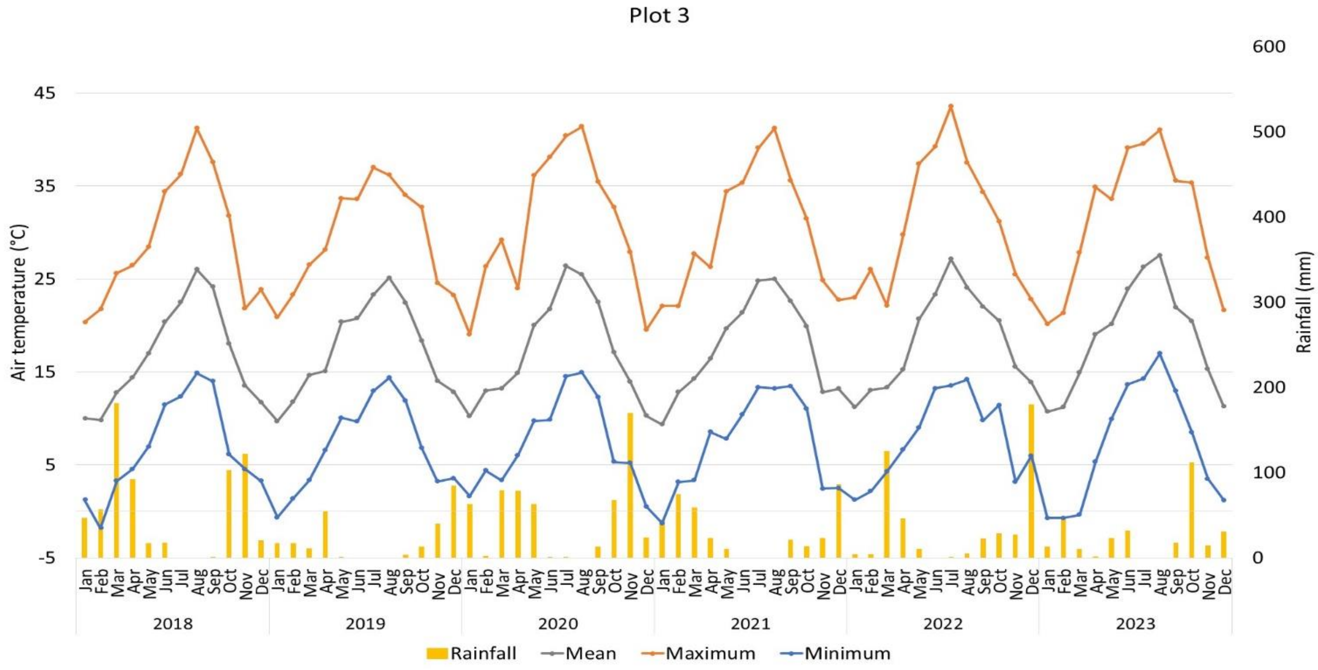

Figure S1. Monthly minimum (blue, °C), mean (grey, °C) and maximum (orange, °C) air temperature, rainfall (yellow, mm) registered in the three different plots from 2018 to 2023.
